# Professional educators’ experiences teaching internationally educated nurses attending bridging programs: a qualitative interview study

**DOI:** 10.1186/s12912-025-03414-0

**Published:** 2025-06-27

**Authors:** Anna-Maria Sarstrand Marekovic, Emina Hadziabdic, Päivi Juuso, Kristiina Heikkilä

**Affiliations:** 1 Department of Social Studies, Faculty of Social Sciences, Linnaeus University, Växjö, SE-351 95 Sweden; 2 Department of Health and Caring Sciences, Faculty of Health and Life Sciences, Linnaeus University, Växjö, SE-351 95 Sweden; 3 Department of Health, Education and Technology, Luleå University of Technology, Luleå, SE-971 87 Sweden; 4 Department of Health and Caring Sciences, Faculty of Health and Life Sciences, Linnaeus University, Kalmar, SE-391 82 Sweden

**Keywords:** Bridging programs, Internationally educated nurses, Qualitative research, Recertification process

## Abstract

**Background:**

To aid in the recertification and integration process of internationally educated nurses (IENs), many countries have organized bridging programs to provide support and education and align their knowledge and competence with local requirements. The importance and relevance of these programs for IENs are highlighted in related research. However, the experiences of university teachers, supervisors in clinical placement, and administrators organizing these programs are scarcely explored. The aim of this article is to explore the experiences of professional educators engaged in bridging programs at two universities in Sweden.

**Methods:**

A descriptive qualitative approach was employed. The data were collected between December 2021 and March 2023 through semi-structured interviews with 15 educators involved in bridging programs at two universities in Sweden. The participants included university teachers, administrators, and supervisors in clinical placement. The data were analyzed using thematic analysis.

**Findings:**

The thematic analysis identified two main themes: (1) teaching a heterogeneous group of students and (2) organizational constraints of bridging programs. In relation to the first main theme, three subthemes were identified: educational and professional heterogeneity of IENs, varying language competencies of IENs and finding new approaches to teaching IENs. For the second main theme, three sub-themes were identified: compressed time in bridging programs, lack of resources for a time-consuming education and bridging programs as competition for resources.

**Conclusions:**

This study contributes to the understanding of the challenges associated with the recertification process of IENs through bridging programs from the perspective of educators. The findings highlight that educating a heterogeneous group of students, such as internationally educated nurses with differences in professional experience, knowledge and local language competencies, is time-consuming. Educators need to learn about the level of knowledge of the student group and individual learning needs and adapt the instructions accordingly, which is constrained by the organization of the programs. The findings also indicate that educators can find ways to mitigate these challenges and adapt their instructions to meet the needs of students. Therefore, it is important to allocate time and resources to running bridging programs and to support the cooperation and exchange of information between universities and clinics. We also suggest the need to increase the competency level of educators on how to support learning through a second language and enhancing collaboration with language instructors to ensure high-quality education for IENs.

**Supplementary Information:**

The online version contains supplementary material available at 10.1186/s12912-025-03414-0.

## Background

Nurses have been migrating internationally for a long time, and this trend continues to grow. This is primarily due to a shortage of nurses in many developed countries, which are seeking to fill gaps in their healthcare workforce [1, 2]. In the OECD area, the number of foreign-trained nurses rose by more than 20% between 2011/12 and 2017/18, though the numbers vary between countries. Australia, Canada, and the United States continue to have the highest number of foreign-trained nurses, while countries such as Switzerland, France, and Germany have experienced particularly rapid increases in nurse migration. Sweden has also experienced an increase in nurse migration, where the number increased by 15% in five years [3]. However, global differences in healthcare systems and professional standards pose challenges for the smooth transition and professional re-entry of internationally educated nurses (henceforth, IENs), i.e., individuals who have completed a nursing education program outside the country where they seek employment [4]. To address this, many countries have implemented validation processes and bridging programs to align knowledge and competence with local requirements [5, 6]. The organization and content of these programs vary among countries but all aim to aid in the recertification and integration process by providing support and education [7, 8]. In Canada, for example, the credential assessment for nurses is regulated by professional associations [9], while in Sweden the national agency the Swedish Board of Health and Welfare is responsible [10]. Further differences include the length of the bridging programs ranging from a few months up to several years [8, 11, 12]. In Sweden, bridging programs for IENs include one year of full-time studies encompassing theoretical and practical courses. The programs are intended to build on IENs’ existing education and knowledge rather than requiring them to undertake an entirely new university degree, thereby facilitating their ability to secure employment that aligns with their qualifications [13].

Research highlights the importance of recertification programs for IENs in facilitating their return to nursing and adaptation to host country standards [14, 15] by helping them understand the roles and responsibilities of nurses in the new context [7, 11, 16]. Furthermore, these programs have proven to be successful at fuelling participants’ desire to reestablish themselves as nurses and providing academic and practical support in the recertification process [10, 12, 14]. Participation in bridging programs has contributed to students’ self-reported improvements in self-esteem and confidence [17]. However, these programs present challenges, such as being time-consuming and economically burdensome for IENs [18, 19]. The value of bridging programs for host societies has been explored in research highlighting their contribution to the diversity of the nursing workforce, thus meeting all patients’ needs, specifically those of ethnic minorities [20]. Cruz et al. [11] conclude that “Putting in place bridging programs that meet both the IENs’ and host country’s needs may prove to be an essential tool to facilitate IENs’ return to nursing” (p. 1108).

Despite their importance, research on the organization of bridging programs and the experiences of professional educators such as university teachers, supervisors in clinical placement, and administrators involved is limited. These actors play crucial roles in supporting IENs’ professional development and assessing their qualifications as nurses [12, 21]. There are a growing number of studies on the supervision of IENs and culturally and linguistically diverse nursing students (CALDs). Although these two student groups differ in where they obtained their education – either within or outside the employer country – research highlights similarities in the rewarding yet challenging nature of supervising them, as experienced by educators. Challenges include a lack of education in intercultural communication, which could be an important tool to bridge the linguistic and cultural divide between supervisors and students [22, 23]. The need for increased support and collaboration with higher education institutions is emphasized to better identify learning needs and adapt mentoring to varying levels of competence [24–26]. Further research is needed to expand the knowledge on bridging programs from the perspective of key stakeholders, including university teachers and administrative staff.

This study is part of a broader research initiative examining the recertification and professional re-entry process for IENs in Sweden. While much of the previous related research has focused on the experiences of IENs themselves, there is a gap in knowledge regarding the perspectives of educators in bridging programs. This study aims to fill this gap by exploring the experiences of professional educators engaged in bridging programs at two universities in Sweden. By identifying challenges and rewards in teaching IENs attending bridging programs, this research contributes to the advancement of these programs.

### The recertification process for IENs in Sweden

Unlike many other host countries, such as the UK, US, and Canada, Sweden has not actively recruited IENs to address its nursing shortage. Instead, Sweden has drawn on the spontaneous migration of individuals with nursing qualifications through other channels, such as family migration, labour migration, or refugee migration, which has contributed to the diversity of the population [9, 19, 27]. However, the requirements for IENs to practice as registered nurses in Sweden vary depending on the country where their education was completed. Nurses educated in Nordic countries face no barriers to employment, whereas those moving within EU countries must meet Swedish, Norwegian, or Danish language requirements and provide proof of formal recognition of their professional qualifications to obtain a license from the Swedish Board of Health and Welfare [28].

Nurses educated outside the EU/EEA who seek employment in Sweden must first have their foreign nursing degree validated by the Swedish Board of Health and Welfare, confirming that it is formally recognized as sufficient for pursuing licensure in Sweden. This is followed by proof of proficiency in Swedish equivalent to level C1, which indicates that the individual has advanced language proficiency [29]. Thereafter, the person can choose to undergo a series of individual examinations or apply to a bridging program for further education before applying for a Swedish nursing license [6, 14]. A recent study by Hadziabdic et al. [30] shows that most IENs migrating to Sweden from outside the EU/EEA and pursuing recertification through bridging programs come from Asia, the Middle East, and Africa. The study also finds that their primary reasons for migration are family reunification or refugee status. As a result, IENs in bridging programs represent a highly diverse group in terms of prior education, national origin, migration background, and age, though they are predominantly female.

The initial Swedish bridging programs for IENs commenced in 2009 at two universities and later expanded to three more universities. These programs encompass one year of full-time coursework, ideally tailored to each student’s background [14]. The curriculum elements include theoretical modules on nursing practice, pharmacology, leadership, and practical courses in clinical placement across various healthcare settings. Program structures may vary across institutions due to the absence of national regulations. Upon completion, IENs must pass a knowledge examination administered by the university offering the program. This examination assesses theoretical and clinical competencies equivalent to those required in undergraduate nursing education programs and is a prerequisite for applying for licensure through the Swedish Board of Health and Welfare [10]. This process typically spans 1–3 years [6]. University personnel, including teachers and program directors, are pivotal in delivering theoretical education. Clinical placement supervisors guide students in clinical practice, facilitating discussions and providing feedback on nursing practices and on students’ progress and learning [26]. Both actors assess IEN course work.

## Methodology

We employed a descriptive qualitative design [31] to explore educators’ experiences and identify challenges and rewards in the recertification process. Thematic analysis, inspired by Braun and Clarke’s [32] framework, was used for data interpretation following a hermeneutic approach [33]. This approach facilitated a deeper understanding of the participants’ experiences and the meanings they ascribe to teaching IENs, providing valuable insights for improving and advancing bridging programs [33].

### Setting and participants

Access to the field and recruitment of research participants for this study began in December 2021 and continued through March 2023. This period partly coincided with the COVID-19 pandemic, which may have impacted both the response time and the participation rate. During this time, university teachers and nurse practitioners faced an especially intense workload [34, 35], likely making them even more stressed for time than usual. First, we contacted two universities offering bridging programs, seeking consent to engage participants. We were then provided contact information for relevant individuals involved in the bridging programs, including program directors, university teachers, administrators, and supervisors in clinical placements. In total, the list included 55 individuals. We began by sorting out individuals with little involvement in the programs, such as general administrators and coordinators working at regional councils, after which the list included 34 persons. The first author then proceeded to contact the potential participants by email or telephone, presenting information about the study and the purpose of the interview. Some individuals did not respond to our inquiries, whereas others declined due to time constraints, their limited experience in teaching or supervising IENs, or because it had been several years since their last involvement. This phase of the research process was prolonged and involved waiting for responses and recontacting potential participants. At the end of the first year, we reached out to our gatekeepers at the two universities, who assisted us in re-disseminating information about the project. This renewed interest among participants, allowed us to complete the final interviews. At this stage, as the interviews provided rich descriptions, we concluded that we had reached data saturation, as no new information was reported by the participants [31].

In total, we received positive answers from 15 individuals representing university teachers, including one program director (7), supervisors in clinical placement (6) and coordinators for clinical placement (2), who were evenly distributed between the two universities. All participants, but one, were female. Both the university teachers and the supervisors held nursing degrees and had experience in clinical practice. The university teachers and program director had between two and three years of experience teaching IENs in bridging programs, and all had several years of experience teaching in undergraduate nursing education programs. The clinical supervisors had mentored between one and three IEN students, and all had experience supervising students in undergraduate nursing education programs. The coordinators for clinical placement had administered the bridging program for two or three years (see Table 1).

**Table 1:** Characteristics of the study population

Variable	Persons (*N*= 15)	
Gender *(n)*		
	Female	14
	Men	1
Age (years)		
	29–39 years	2
	40–49 years	5
	50–59 years	2
	60–65 years	6
Work role *(n)*		
	University teacher	7
	Clinical supervisor	6
	Coordinator for clinical placement	2
Years in current role		
	1–5 years	5
	6–10 years	3
	11–15 years	3
	16–25 years	4

### Procedure and data analysis

Knowledge and findings from our previous study [10], in addition to existing research on the recertification processes for IENs [6, 7, 15, 22, 28] informed the identification of key aspects to explore regarding the experiences of professional educators. In particular, this body of work highlighted issues related to program structure and content, students’ prior knowledge and educational needs, and their integration into both the educational system and the labour market. Based on these insights, we developed a semi-structured interview guide focused on three main areas: (1) participants’ roles and experiences in the bridging program, (2) perceptions of the program and students’ knowledge and needs, and (3) challenges and opportunities for IENs in Swedish healthcare. Open-ended questions allowed participant reflection, with follow-up questions for clarification and exploration of individual variations. The interview guides were slightly adapted to the different roles of the educators (see Supplementary material 1 and 2). The interviews were conducted individually in Swedish by the two researchers with experience in conducting qualitative research on migration issues. They lasted between 23 and 78 min (average: 46 min) and were conducted primarily via Zoom videoconferencing (13), by telephone (1), and, in one case, face-to-face.

All the interviews were audio-recorded, transcribed verbatim, and analyzed thematically, drawing on Braun and Clarke’s approach [32]. Thematic analysis is a flexible method that allows for a detailed description of data and the identification and reporting of meaningful patterns. Although it shares similarities with other qualitative research approaches, thematic analysis specifically guides the active identification and reporting of themes, that is, patterns of shared meaning in the data [32]. We employed an inductive iterative analytic method, starting with the data and guided by the research aim. The first author began by reading all transcripts to familiarize herself with the data, making notes on initial ideas and interpretations. She then proceeded with initial coding and sorting of the codes into two preliminary themes. These preliminary themes were presented to the research group, who collectively discussed and reviewed them, which led to the identification of additional patterns in the data. These patterns were then further explored by the last author, who returned to the transcripts to review the analysis. This iterative process prompted a re-coding of the dataset, during which the main themes were revisited, and sub-themes were identified which enhanced the organization and structure of the thematic framework. The re-coding and the development of themes and sub-themes were conducted collaboratively by the first and last authors. This phase resulted in two main themes, each comprising three corresponding sub-themes. The refined thematic structure was then presented to the second and third authors, who provided critical feedback and contributed to further discussion and refinement of the analysis. Throughout this process the codes and themes were continually reviewed to ensure internal homogeneity and external heterogeneity [32].

### Ethics

Ethical vetting was sought and approved by the Swedish Ethical Review Authority (No. 2021–03844). All participants were given written information about the project and the purpose of the interview when asked about their participation. The information was repeated verbally at the time of the interview, with information about the right to withdraw from the study at any time, ensured informed consent from all participants. To protect the confidentiality of our research participants, we opted to categorize them broadly as ‘educators’, which includes university teachers, program directors, clinical placement coordinators and clinical supervisors.

## Findings

The analysis identified two main themes: teaching a heterogeneous group of students and organizational constraints of bridging programs, which convey the educators’ experiences of teaching IENs attending bridging programs. With respect to the first theme, three subthemes were identified: educational and professional heterogeneity of IENs, varying language competencies of IENs and finding new approaches to teaching IENs. For the second main theme, three sub-themes were identified: compressed time in bridging programs, lack of resources for a time-consuming education and bridging programs as competition for resources. The themes and sub-themes are presented in the table below (see Table 2).

**Table 2:** Thematic analysis of professional educators’ experiences teaching IENs

Main theme	Sub-themes
1. Teaching a heterogenous group of students	a. Educational and professional heterogeneity of IENs b. Varying language competencies of IENs c. Finding new approaches to teaching IENs
2. Organizational constraints of bridging programs	a. Compressed time in bridging programs b. Lack of resources for a time-consuming education c. Bridging programs as competition for resources

### Teaching a heterogenous group of students

The first main theme highlights the educators’ experiences and perceptions of teaching IENs attending bridging programs. The educators noted that IEN students have distinct needs and prerequisites compared with those in undergraduate nursing programs, requiring them to develop new teaching approaches.

#### Educational and professional heterogeneity of IENs

The educators described challenges teaching IENs due to the diverse educational backgrounds of the students, which vary in duration and emphasis on specific nursing skills across different countries. Additionally, the students’ prior nursing experience ranged from minimal to several years, further complicating the educational process. Moreover, their experiences in Swedish working life varied, while some students had extensive experience as assistant nurses in elderly care for several years, others had little to no prior work experience in Sweden.

They come from such different contexts, and how nursing education is organized in different countries can vary greatly […]. Some of them had not worked as nurses, and others had worked for a truly long time and had specialist education. Therefore, the group is very diverse in that way; some have a lot of experience, and others have none. To try to fit everyone in the same structure and obtain some kind of uniformity in one year…it is truly difficult. (Educator 8)

These differences pose challenges in planning and designing programs and courses, ascertaining students’ specific needs, and supplementing their education to meet Swedish nursing standards. The heterogeneity in educational backgrounds, working life experiences and nursing skills fostered a sense of uncertainty among the educators about whether the students were being equipped with the skills required for nursing practice in Sweden. The educators consistently reflected on their responsibility to ensure that all the students acquired comprehensive competencies, encompassing both professional and clinical aspects of nursing. The practical clinical skills of IENs are often emphasized and regarded as an asset in the context of Swedish healthcare: “[…] They possess a lot of knowledge, that we can benefit from. More importantly, they have so much experience that they bring to our patients here” (Educator 2). However, educators expressed concerns about the challenges many IENs face in adapting to the culturally specific requirements of nursing in Sweden, such as leadership, professional autonomy, and collaboration with other healthcare professionals.

What I’ve found challenging [in teaching IENs] is the responsibility we have as nurses. It’s about making assessments, not just calling the doctor to report. I’ve seen it quite often that they do everything right, gather all the information, but don’t take a stand on it. The solution is to call the doctor to tell them what to do. That’s not how it works in Swedish healthcare. […] Therefore, we’ve had to work on that and truly emphasize the importance of making our own judgments. (Educator 7)

Several of the educators described the process of adapting to the Swedish nursing role as developing over time, including for students in the undergraduate nursing program. However, in the context of the bridging program, this had to continue quickly, and the educators described how most IENs struggled with this.

#### Varying Language competencies of IENs

The educators noted the diversity within the student group in terms of language skills. All instructional materials and courses in the programs were delivered in Swedish, with proficiency in the language a prerequisite for admission to ensure that all the students met a specified language competency level. However, during the interviews, all the educators reported observing varying levels of Swedish proficiency among the IENs and how this proficiency affected their educational activities. While some students had no difficulty expressing themselves verbally or in writing, others were assessed as having insufficient Swedish skills. The deficit not only impacted the students’ ability to complete theoretical courses but also affected their assessment of their clinical placement. Language barriers and misunderstandings during clinical placement were described as potential risks to patient safety, as emphasized by Educator 1, who stated, “When there are significant language barriers, it impacts patient safety too, a crucial aspect when assessing students”. Moreover, supervision was described as more challenging due to the diverse range of language skills among students and the associated risks. The supervisors found themselves needing to dedicate extra time and effort to thoroughly reviewing the work of IENs, which included assessing their written documentation and patient communication, to mitigate potential misunderstandings that could jeopardize patient safety. Insufficient language proficiency among IENs fostered a sense of uncertainty among educators regarding the students’ actual grasp of nursing practices, making assessment more challenging. Simultaneously, IENs were described as an enthusiastic student group that was easy to engage in discussions and eager to learn, which was a positive experience for the educators.

I think that this group, and I’ve also had that confirmed, is a fun group to meet. […] It’s this commitment, and you can hear that there is a passion for the profession itself and a desire to contribute and be involved. In addition, I believe they bring professional pride as well. (Educator 8)

The educators expressed appreciation for working with a culturally diverse group of students. The students’ questions and narratives of their countries’ health care system opened up their eyes and challenged their taken-for-granted ways of doing things, thus forcing them to think in new ways. Although challenging, this was a reward for the educators, as it made the work interesting.

#### Finding new approaches to teaching IENs

To manage the heterogeneity of the student group, educators developed new approaches to student education to better meet the needs of the student group. One area pertained to the diversity in the educational and professional experiences of the IENs, which made planning courses difficult. To manage this, the educators developed a strategy to learn more about the student’s experiences and knowledge at the beginning of a new course. By gathering that information, the university teachers could adapt the content of the course to the learning needs of the specific group.

Well, we know that they are educated nurses, so I usually start the first lesson by checking with the group, asking about their experiences and how much medication they have handled, and such. Just to have an idea about who I have in front of me. And everyone has handled medications in some way because they have received education. However, it still varies from country to country in terms of what they are allowed to do and such. (Educator 5)

The strategy that the educator describes in this excerpt gives her an idea of the heterogeneity of the group in relation to a particular course, which makes it easier to prepare and adjust the content to the needs of the group. This strategy included a need for flexibility as they needed to modify their planned course structure and content in short notice. The fact that the student groups attending bridging programs at the two universities were often modest in size, generally between 15 and 20 students, allowed the teachers to maintain this flexibility.

Similarly, the supervisors described the importance of learning more about the IEN students’ background, experiences and learning needs to adapt the clinical placement accordingly.

I’m looking at what their learning objectives are, what they have done before, what they themselves think they need to strengthen, and what they already feel like ‘I don’t need to put much effort into this’. To get a picture of them and at the same time get to know them a little. (Educator 11)

The importance of knowing about the student’s background and experiences is highlighted in this excerpt, as this gave the supervisors some clues to which areas they needed to focus on. However, in clinical placement, adapting approaches to individual needs was easier, as the students had designated supervisors who could check the knowledge and skills base of each individual student and maintain regular communication throughout the placement period.

Another area that needed adapted teaching practices concerned communication and Swedish language skills. One strategy to aid the students in becoming comfortable with Swedish health care vocabulary and strengthening their skills in Swedish, as a part of becoming Swedish nurses, was to offer more teacher-led instructions than was provided at the undergraduate nursing education program. Meeting with the students more regularly was described as one way to enhance their comprehension of Swedish, as there was more time to ask questions and discuss the course content. At one of the universities, two of the teachers had regular follow-up sessions with the IENs to address misunderstandings or to expand on the course content.

The educators also adapted their presentation style to use wording that was more accessible to the students or to actively vary how they explained different course elements. One educator said:

It feels like I almost change my tone and speak more slowly and clearly when I meet them, just so that they can understand. And [I] ask quite often, ‘Do you understand, or do you have any questions?’ (Educator 5).

This strategy was used not only in oral communication but when questions were posed during examinations. In addition, the educators included activities with the aim of increasing the students’ language proficiency through discussions and reflections, for example, exchanging experiences of nursing work in different national contexts or reading a Swedish novel. In clinical placement, the possibility of one-on-one education could be adapted to target students’ language challenges. Educators could advise students to expand their medical vocabulary by pointing them to relevant literature or online sources. However, they also used direct strategies aimed at supporting the IENs’ language development. One educator described how she was able to monitor and give specific instructions on journal documentation: “It was mainly about how to write, how to formulate oneself, which keyword to use… Yes, I think you had to spend a bit more time on that” (Educator 4). Some of the educators mentioned that they sometimes advised individual students to contact the writing center at the university library to obtain assistance and help improve their skills. However, this approach was not performed regularly or implemented as part of education at either of the universities. One educator reflected on the need to support the students’ linguistic development: “It’s actually two educations that these students are undertaking. They are doing a language education and a vocational education. […] I think, we probably should have put more effort into that” (Educator 13).

### Organizational constraints of bridging programs

The second main theme identified the educators’ experiences with the organizational challenges of bridging programs and their insights into how these programs could be developed and improved.

#### Compressed time in bridging programs

One challenge that was described was the absence of national guidelines governing bridging programs. Each university enjoys autonomy in structuring the program content and determining the specific courses offered but must include theoretical courses as well as clinical placement courses to assess the students’ competencies. While this combination of courses was recognized as essential for catering to the diverse needs of the students and addressing various aspects of nursing practice, it resulted in the perception of program fragmentation. As Educator 1 said, “It’s like a smorgasbord, and it can seem a bit rhapsodic at times”.

A consequence of the perception of the program as fragmented was that many educators expressed that the one-year program was too short for covering all relevant aspects of nursing and for the students to comprehend the new professional context: “It’s difficult to squeeze three years [education] into one year. Sometimes it feels a bit like cramming, because we don’t have enough time.” (Educator 9). The content and structure of the programs were ultimately a result of trying to balance different needs. Educators observed that the intensive pace of the program often translated into heightened levels of stress among students, who struggled to juggle their educational commitments alongside other responsibilities: “It is very stressful for the students to have many tasks tightly scheduled. In addition, many of them have families and jobs too” (Educator 9). The quote from the educator illustrates an awareness of the life situations of many of the students, which often included family responsibilities and the need to provide resources economically for them, impacting the duration of their studies. The time in education was regarded as too limited in relation to the learning objectives of the program.

I think one year isn’t enough. It doesn’t have to be three, but perhaps at least a year and a half. […] I feel like we don’t have the time to cover all that we need to fulfil the requirements of society and the National Board of Health and Welfare. However, also, for the students to ensure they feel confident in their abilities, especially in the Swedish context. (Educator 7)

The compressed time in education made it difficult to cover the different learning content and ensure that the students were prepared for working life. In particular, clinical placement courses were seen as essential for IENs to adapt to the Swedish context. The educators expressed a need for more and longer courses as part of the program to give the students time to integrate theoretical and practical knowledge and lower the stress of the students.

[It] would have been good to extend certain clinical placements. And restructure it so that students could go out to their first placement even earlier, because the important thing is that they get early contact with the field they will eventually work in, while they are studying the theory. (Educator 1)

#### Lack of resources for a time-consuming education

Furthermore, the educators reported that working in bridging programs was time consuming and that educating IENs was more demanding than educating nursing students in the undergraduate program. This experience is a consequence of the heterogeneity of the student group, which demands more individual guidance. For the university teachers, it became apparent that the time they needed to devote to the courses and the student group well exceeded the number of working hours that were allocated to the bridging program.

All of us involved in the program felt that…there was no time for it. It was like we were expected to do this with our left hand. Even our managers shared this view. They didn’t grasp the extent of the effort we actually had to invest in these students. (Educator 13)

Additional time was often needed to explain in greater depth the content of the courses and to respond to the many questions from the students. Similar experiences were reported by the clinical supervisors, who often devoted more time to supervising students from the bridging program. One reason that was reported was that the knowledge needs differed between the students, and the supervisors needed time to determine the specific areas of support for each individual student. The time it took to make this decision varied and was longer when educators had difficulty assessing the student’s competency level. In such cases, closer supervision during clinical placement was needed, which demanded more time.

I could never let that student work on her own; I was always like a shadow behind her because I didn’t want to put the patient or the staff in a situation where they felt unsafe or like ‘something’s happening here that I can’t support.’ So, she never went to the patients on her own, I was always there, even if I didn’t always intervene, just listening or monitoring the situation. (Educator 11)

#### Bridging programs as competition for resources

Organizational constraints related to competing interests, especially in relation to the undergraduate nursing education program, were repeatedly reported by the educators. One area that was frequently discussed was clinical placements. The difficulty of finding enough placements is common to all nursing programs– undergraduate as well as specialist education– but the placements of IENs are sometimes described as happening at the expense of placements for students in undergraduate programs.

You know, they already accept quite a few students. Therefore, when this additional group came, we might have had to replace a student from semester six [of the undergraduate nursing program] so that they could take this one [IEN] instead. […] Therefore, we perhaps had to remove some other student to make room for these. (Educator 6)

In this way, the education of IENs contrasted with the education of other nursing students. The extra resources and time needed to educate IENs were reported as obstacles that made workplaces hesitant to accept IENs for clinical placement. Considerable work and discussion was needed for representatives of bridging programs to convince clinics of the benefits of accepting IEN students.

We were actually met with quite a lot of scepticism from our stakeholders, where they focused more on the problems than the possibilities. I mean, it’s a huge opportunity to have a fully qualified nurse in just one year. (Educator 1)

## Discussion

This study contributes to the growing body of research on the experiences of nursing educators, both academic and clinical, regarding the instruction of internationally educated nurses in bridging programs. The findings reveal that there are challenges associated with these programs that relate to the composition of the student base and the organization of the programs. Moreover, the findings indicate that educators explore and find ways to mitigate challenges and adapt their instructions to meet the needs of students. Their reported experiences indicate that bridging programs involve “two educations in one”. First, professional education is needed to ensure that IENs have knowledge and competence comparable to the national requirements. Second, linguistic and cultural education is needed to prepare IENs for communication and interaction in local healthcare settings. This dual purpose of the program posed challenges to the educators that needed to be managed. Although they felt somewhat uncertain in managing the heterogeneous student group, they were stimulated by the experience and learned to grow with the challenges. These results are supported by findings in previous research, highlighting that educating IENs is filled with both frustration and joy [26].

The analysis identified experiences of teaching a heterogeneous group of students. IENs differ in educational background, prior nursing experience, and emphasis on nursing skills across countries. These differences created a sense of uncertainty among the educators of the students’ prior knowledge and skills and thus about what the education must equip them with to meet Swedish requirements. The analysis identified that the educators were not at loss to the educational situation but developed flexible approaches to meet the diverse needs of students, including adjusting course content on the basis of individual and group circumstances. This adaptability was facilitated by the modest size of the student groups, allowing for personalized attention and the possibility of adjusting the course content or clinical placement to specific needs of the group. Similar findings were reported in several studies of clinical supervisors’ experiences [22, 24, 26] as well as teachers’ [21] involvement in the education of IENs or CALD nursing students, indicating the need to learn about the students’ backgrounds and the importance of sharing this knowledge among all the actors involved in the education. Eriksson et al. [26] emphasized the need to improve communication between university teachers and clinical supervisors prior to student placement to better prepare supervisors, which our findings support. We suggest that the diversity of IEN students be addressed through adapted mentoring programs, both in the theoretical and practical parts of the education, thereby enabling the identification of individual prior knowledge and experience, assisting in the organization and planning of the program activities and supporting the assessment of existing skills. In line with Mikkonen et al. [36] and Ruvang et al. [37], we propose that this preparation could include intercultural competence and information on nursing work in different national contexts. This approach would not only facilitate teaching and supervision but also better adapt the education to the individual needs of the student, which in turn can ease their professional integration.

Another significant challenge for educators is the variation in IENs’ proficiency in the local language, which often leads to uncertainty in both instruction and assessment. Language barriers are widely recognized in the literature as obstacles to learning and can contribute to negative attitudes toward these students [23, 38]. Although most studies on this issue stem from English-speaking contexts [39, 40], the challenges are likely even more pronounced in countries with linguistically complex or less commonly taught languages, such as those in Scandinavia Lum and Vu [41] found that in English-speaking countries, IENs with pre-migration English proficiency integrated more successfully into the labour market than those who acquired the language after arrival. Given that Swedish is a relatively minor global language, few IENs – or immigrants more broadly – are likely to have prior knowledge of it, which may hinder their educational progress and increase demands on educators. similar to Lum and Vu [41], our findings emphasize the importance of incorporating IENs’ pre-migration competencies – particularly language skills – into the design of bridging programs to better support both students and educators. This is further reflected in our findings, which show that when a student’s language competency is questioned, educators often feel uncertain about the students’ practical clinical and nursing skills, making assessment a critical task. As in previous studies [24, 26], educators in our study reported that many students lacked sufficient proficiency in the local language, despite having passed the required language courses to enter the program. Our study adds to this by showing how educators developed strategies to support students’ language development, including providing more teacher-led instruction, adapting presentation styles, and creating more opportunities for language practice and support. As a result, the student group demands additional resources from educators, a need also emphasized by Eriksson et al. [26] and Juntunen et al. [21]. In response to these challenges, some Swedish universities have introduced a mandatory semester of language training prior to bridging programs [26]. While this can effectively strengthen students’ language skills, it imposes additional costs on both students and institutions. A complementary strategy is to enhance educators’ competence in second language learning, which could support their professional development, improve communication with students, and strengthen overall program quality [28]. Similar benefits may be achieved through closer collaboration between nursing educators and language teachers, integrating theoretical and practical courses with targeted language instruction, as suggested by Cubelo et al. [42].

As indicated above, the present study revealed that all participants expressed that educating IENs is more time-consuming than educating students from undergraduate nursing programs. For example, educators described communication problems, low language competency, and cultural differences as issues that needed to be managed; these issues required close monitoring of the student and hence more time [21, 39]. This was stated as a reason to be hesitant to accept IENs for clinical placement. These findings highlight the importance of offering additional support and more detailed information about each student’s learning needs to clinics and prospective supervisors for successful clinical placement of IENs. In that way, supervisors can feel more prepared and adapt the practice to the individual student. Similarly, university educators invest significant additional time to ensure that students understand course content and adapt their teaching to the needs of diverse student groups. As a result, many reported that the time allocated to bridging programs is insufficient, given the hands-on support required to guide IENs in becoming Swedish nurses and understanding key aspects of Swedish healthcare culture, including leadership, professional autonomy, and interprofessional collaboration. These findings align with those of Juntunen et al. [21] and Mikkonen et al. [37], who observed that teaching CALD students demands considerable extra time. To improve working conditions for educators and ensure high-quality education for IENs, increased resource allocation to bridging programs is essential, as also emphasized in previous research [21, 24, 26].

### Limitations and future research

A limitation of our study was the exclusion of a considerable number of participants who either declined or were unreachable. As discussed in the Methodology section, one challenge in recruiting participants was the concurrent COVID-19 pandemic, which likely limited opportunities for engagement [35, 36]. A second attempt to recruit participants, initiated after the pandemic, allowed us to complete data collection. This experience underscores how external stressors – such as public health crises – can significantly affect researchers’ ability to conduct qualitative studies, particularly those requiring close interaction and participation.

Although we can speculate only on the reasons for declining participation, there is a risk that some perspectives and experiences of educators were not captured in the final analysis. This introduces a potential selection bias in our sample, which may have influenced the findings. However, interviewed participants in various educational roles and with experience from bridging programs at two different universities contributed to the diversity of our sample. Despite this diversity, no significant discrepancies in experiences were identified between the two settings or among the different roles, suggesting the robustness of our data collection and analysis. However, with a larger and more differentiated group of participants, more role-specific insights may have been identified. Future research should explore IENs’ transition into the workplace, including their integration into new healthcare settings and their reception by key stakeholders, to further inform the development of bridging programs.

## Conclusions

This study contributes to the understanding of the challenges associated with the recertification process of IENs through bridging programs from the perspective of educators. This perspective is neglected in extant research, which has mainly focused on the experiences of IENs. The findings highlight that educating such a heterogeneous group of students as IENs, with differences in professional experience, knowledge and skill base, can be both frustrating and time consuming. It is therefore imperative to allocate additional time and resources to delivering these programs. Educators need to learn about the level of knowledge of the student group and individual learning needs and adapt the instructions accordingly. There is also a need for resources to support the cooperation and exchange of information between universities and clinics. This could prepare supervisors and thus ease the perceived burden of supervising IENs. The study also highlighted the variation in competency in the local language among IENs and how educators struggled to adapt their teaching. Given that both verbal and written communication and documentation are crucial parts of the nursing profession, ensuring that students have the required skills is necessary. Organizing mandatory language courses before education entry is one way of securing the necessary skill base. As this may be costly, we suggest strengthening educators’ understanding of how to support learning through a second language and enhancing collaboration with language instructors to ensure high-quality education for IENs. These measures can help build institutional support, reduce the burden on individual educators, and ultimately enhance the overall quality of bridging programs.

In conclusion, our study contributes to the growing body of international research on IEN recertification processes and educational conditions, highlighting challenges common to all countries and contexts. A common issue internationally is how to effectively recognize, transfer, and align IENs’ diverse skills and competencies with national standards and regulatory frameworks, which is often a complex and resource-intensive process, as confirmed by our study. Importantly, our study also adds to the understanding of challenges specific to non-English-speaking countries like Sweden, where the linguistic dimension is particularly pronounced. The findings emphasize the need for bridging programs to more explicitly address language-related barriers in order to facilitate IENs’ successful transition into the local healthcare workforce.

## Electronic supplementary material

Below is the link to the electronic supplementary material.

Supplementary Material 1

Supplementary Material 2

## Data Availability

The data sets used and/or analyzed during the current study are available from the corresponding author upon reasonable request.

## Abbreviations

IENs: Internationally educated nurses
CALD: Culturally and Linguistically Diverse Nursing Students
