# Supplementary material for: Professional educators’ experiences teaching internationally educated nurses attending bridging programs: a qualitative interview study
Source: BMC Nurs. 2025 Jun 27;24:672. doi: 10.1186/s12912-025-03414-0 (PMC12203720; doi:10.1186/s12912-025-03414-0)
Supplement: Supplementary file 1 — Supplementary Material 1 [file 12912_2025_3414_MOESM1_ESM.docx]

# Interview Guide 1 (University personnel)

**Background and Primary Work Duties in Relation to the Bridging Program**

- Age, gender, education (profession), work experience.
- Can you describe your main duties within the bridging program (program responsibility, teaching – courses, administration, student contact, etc.)?
  - How much of your role is dedicated to the bridging program? Any other teaching/administrative duties?
- How long have you been working in this role? How did you get this assignment?
- How interested are your colleagues in this education? Are these courses 'popular' to teach?

**About the Bridging Program and Its Participants – Knowledge and Needs**

- What are your views on the program's structure and content?
  - For example, the balance between theoretical and practical (clinical placement) components?
- What structures and regulations govern the program's structure and content? What obstacles and opportunities exist to meet these?
- Tell us a bit about how you perceive the courses/clinical placements are adapted to the target group.
  - What needs do you believe the education meets for this student group?
  - What do you think is missing/needed more in the education?
- What do you perceive that students recognize from their previous education/work experiences, and what is new?
  - In what way do they benefit from their previous work experiences?
- Do you notice differences in the content of the professional role? (e.g., patient-oriented work, care, documentation requirements, independence, hierarchical work relationships, etc.)
- What competencies does this group specifically have? What are their strengths?
  - How do you benefit from the experiences and skills this student group has? Can you give an example? (e.g., professional/formal experiences/competencies and informal competencies such as language/'culture')
- In what way is their multilingualism an asset in teaching? Can you use this skill in any way?
- How do you collaborate with supervisors at clinical placements? What is your contact and relationship like? What works well and less well?
- How is their clinical placement arranged?
- What do you perceive participants struggle with in relation to the education/courses/clinical placement? (specific examples if possible)
- How do you assesse the students' knowledge and how they have achieved the course objectives? Have you had to fail anyone? What caused the person not to be approved?
- What significance do you think language has for students' ability to achieve goals and complete the education?
- What do you think is missing/needed more in the education?
- What do you believe needs to change in the education to better meet the existing needs?

**Challenges and Opportunities for IENs in Swedish Healthcare**

- What do you perceive participants struggle with in relation to the education (both practical and theoretical courses)?
  - Language? How does this manifest in course work? How have you handled deficiencies/needs? (study strategies, language support via the University Library?)
  - Documentation? How does this manifest in your experience? How have you handled deficiencies/needs?
  - Patient-centered work? Contact with patients and relatives? How does this manifest in your experience? How have you handled deficiencies/needs?
  - Medical knowledge and competence/experience? How does this manifest in your experience?
- What needs can this group meet in Swedish health care? Give examples!
- Can you explain the significance of language for the students' ability to achieve their goals and complete their education?
- What adaptations of courses and teaching are made for students in the bridging program?
- With your experience – what is important for succeeding with the education, in terms of passing or maybe more – for the students in the bridging program?
- What do you think this education will lead to in terms of changes in the students' lives?
  - Do you know what happens to the participants after they leave you?
  - What are your thoughts on their future? Education? Work and career?
- What are the most important experiences you take with you from working with students from the bridging program?
- What is the best part of working with this program/participants? What is the hardest/challenging part?
