# Supplementary material for: Professional educators’ experiences teaching internationally educated nurses attending bridging programs: a qualitative interview study
Source: BMC Nurs. 2025 Jun 27;24:672. doi: 10.1186/s12912-025-03414-0 (PMC12203720; doi:10.1186/s12912-025-03414-0)
Supplement: Supplementary file 2 — Supplementary Material 2 [file 12912_2025_3414_MOESM2_ESM.docx]

# Interview Guide 2 (Administrators for Clinical Training Placement & Supervisors)

**Background and Primary Work Duties in Relation to the Bridging Program**

- Age, gender, education (profession), work experience.
- Can you describe your main duties and your role in relation to the bridging program?
- How long have you been working in this administrative role/as a supervisor? How did you come to have this role?
  - How often do you receive students from the bridging program for clinical placement?

**About the Bridging Program and its Participants – Knowledge and Needs**

- How did it come about that you/your organization started accepting students from the bridging program?
  - What attracted you to the assignment?
  - What concerns did you have?
- Can you describe the process when you receive an intern from the bridging program (introduction, people involved, etc.)?
  - How is their clinical placement arranged?
  - How is the introduction to the various tasks at the workplace conducted?
  - Does it differ from how you receive other intern students (from regular programs)?
- What do you perceive that they recognize from previous education/work experiences?
  - In what way do they benefit from their previous work experiences?
  - Do you notice differences in the professional role's content during the internship? (e.g., patient-oriented work, care, documentation requirements, independence, hierarchical work relationships, etc.)
- What competencies does this group specifically have? What are their strengths?
  - How do you benefit from the experiences and skills this student group has? Can you give an example?
  - How do previous work experiences manifest, and how can they be utilized?
  - In what way is their multilingualism an asset in the organization? Can you use this skill in any way? How have you done this (example)?
  - What needs can this group meet in your organization? Give examples!
- How have you assessed the students' knowledge and how they have achieved the clinical placement goals? Have you had to fail anyone? What caused the person not to be approved?
  - What significance do you think language has for students' ability to achieve goals and complete education?
  - What do you think is missing/needed more in the education?
- Tell us how you perceive the education (courses/clinical placement) is adapted to the target group's experiences and competencies?
- How do you collaborate with the education provider at the university? What is your contact and relationship like? What works well and less well?
- What do you think needs to be changed in the education to better meet the existing needs?

**Challenges and Opportunities for IENs in Swedish Healthcare**

- What do you perceive that participants struggle with during the clinical placement and specific tasks?
  - Language? How does this manifest in your organization? How have you handled deficiencies/needs in the intern, patient, or relatives?
  - Documentation? How does this manifest in your organization? How have you handled deficiencies/needs?
  - Patient-centered work? Contact with patients and relatives? How does this manifest in your organization? How have you handled deficiencies/needs in the intern, patient, or relatives? E.g., in relation to communication?
  - Medical knowledge and competence/experience? How does this manifest in your organization?
- What do you do to ease the difficulties that exist? What can you contribute at the clinical placement site?
- With your experience – what is important for succeeding well with the internship/clinical placement for students in bridging programs?
- How did the relationships between the interns and other employees at the clinical placement site work? Difficulties and positive experiences?
- Do you currently or previously have nurses at your workplace who have gone through bridging program? What are your experiences with that?
- If you were to hire a student from the bridging program, what competencies would you look for?
- What are the most important experiences you take with you from working with students from the bridging program?
- What is the best thing about working with this program/participants? What is the hardest/most challenging?
